# Autophagy and epithelial–mesenchymal transition: an intricate interplay in cancer

**DOI:** 10.1038/cddis.2016.415

**Published:** 2016-12-08

**Authors:** Mila Gugnoni, Valentina Sancisi, Gloria Manzotti, Greta Gandolfi, Alessia Ciarrocchi

**Affiliations:** 1 Laboratory of Translational Research, Arcispedale S. Maria Nuova-IRCCS, Reggio Emilia, Italy

## Abstract

Autophagy and epithelial to mesenchymal transition (EMT) are major biological processes in cancer. Autophagy is a catabolic pathway that aids cancer cells to overcome intracellular or environmental stress, including nutrient deprivation, hypoxia and drugs effect. EMT is a complex transdifferentiation through which cancer cells acquire mesenchymal features, including motility and metastatic potential. Recent observations indicate that these two processes are linked in a complex relationship. On the one side, cells that underwent EMT require autophagy activation to survive during the metastatic spreading. On the other side, autophagy, acting as oncosuppressive signal, tends to inhibit the early phases of metastasization, contrasting the activation of the EMT mainly by selectively destabilizing crucial mediators of this process. Currently, still limited information is available regarding the molecular hubs at the interplay between autophagy and EMT. However, a growing number of evidence points to the functional interaction between cytoskeleton and mitochondria as one of the crucial regulatory center at the crossroad between these two biological processes. Cytoskeleton and mitochondria are linked in a tight functional relationship. Controlling mitochondria dynamics, the cytoskeleton cooperates to dictate mitochondria availability for the cell. Vice versa, the number and structure of mitochondria, which are primarily affected by autophagy-related processes, define the energy supply that cancer cells use to reorganize the cytoskeleton and to sustain cell movement during EMT. In this review, we aim to revise the evidence on the functional crosstalk between autophagy and EMT in cancer and to summarize the data supporting a parallel regulation of these two processes through shared signaling pathways. Furthermore, we intend to highlight the relevance of cytoskeleton and mitochondria in mediating the interaction between autophagy and EMT in cancer.

## Facts

- Autophagy and EMT are two major processes in cancer.
- Autophagy plays oncosuppressive function but also serves tumor cells as a strategy to overcome stressful conditions.
- EMT is associated with increased autophagy that helps cells to survive stressful environmental and intrinsic conditions.
- Major signaling pathways including TGF*β* converge on the regulation of autophagy and EMT.
- The reciprocal interaction between cytoskeleton and mitochondria serves as the functional hub in the interplay between autophagy and EMT in cancer.

Open questions

- Which are the molecular mediators that link autophagy and EMT in cancer?
- Which is the role of structural proteins (including Cadherins and Integrins) in controlling autophagy in response to EMT activation in tumor cells?
- How mitochondria dynamics affects cellular architecture during EMT and metastatic spreading?
- How the interplay between autophagy and EMT influences cancer development and progression?

Cancer progression can be regarded as a multistep evolutionary process through which cancer cells acquire competences to survive in severely unfavorable microenvironmental conditions. At each step of progression, cancer cells acquire new abilities to overcome physiological barriers restraining growth. This adaptation process involves alteration of cellular functions and implies cooperation among distinct cellular pathways.^1^

**Figure 1:**
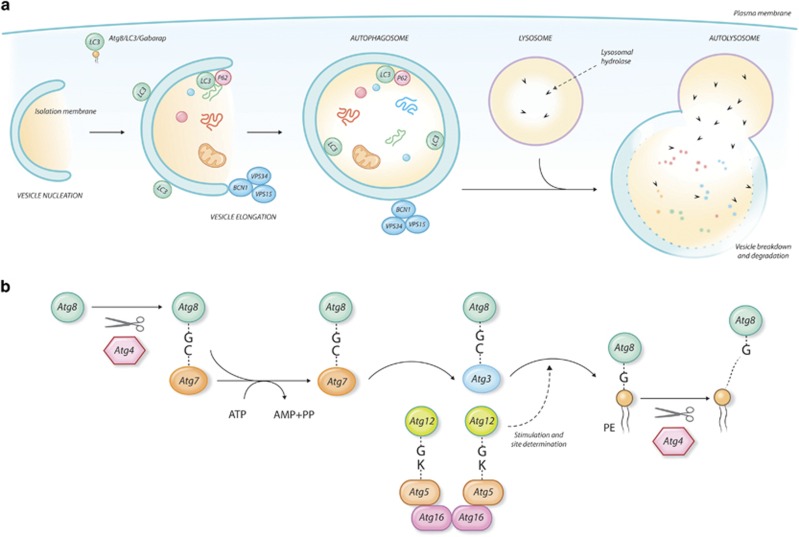
Autophagy. (**a**) Schematic representation of autophagy steps from phagophore formation to fusion with lysosome and degradation of vesicle cargo. Autophagosome is the double-membrane vesicle that loads the cargo to be degraded and fuses with the lysosome to allow digestion. The autophagy machinery comprises a highly conserved set of proteins. In yeast 17 ATG proteins have been identified, many of which have more than one homolog in higher eukaryotes. ATG8 is a ubiquitin-like protein that is loaded on the membrane after conjugation with the membrane lipid phosphatidylethanolamine (PtdEth). This modifies the membrane curvature, triggering the maturation of the phagophore into the mature autophagosome. ATG8 has six homologs in mammals, among which LC3 and GABARAP are by far the most characterized. The ortholog of the yeast ATG6, BCN1 promotes autophagy by interacting with the class III PI3 kinases (VPS34–VPS15) and regulating the positioning of the autophagic proteins to the pre-autophagosomal structures. Autophagy can be non-selective or selective. In the latter case, the cargo is selectively loaded into the autophagosome through the action of specific receptors that mediate the interaction of the cargo with LC3 at the site of forming autophagosome. The first mammalian selective autophagy receptor identified is SQSTM1/p62, an ubiquitin-binding scaffold protein. (**b**) Schematic representation of the conjugation system of ATG8 during the initial phases of autophagy. The loading of ATG8 on the membrane is a multistep enzymatic process that closely resembles the ubiquitin conjugation process. ATG8 proteins are activated by the cysteine protease ATG4 that exposes a C-terminal glycine residue. The processed ATG8 is conjugated with the membrane lipid phosphatidylethanolamine (PtdEth) through the concerted action of the E1 (ATG7) E2 (ATG3) and E3 (ATG12/ATG16/ATG5) enzymes. The E3 ligase complex catalyzes the covalent binding between ATG8 and PtdEth. ATG8 remains associated with the autophagosome until fusion with the lysosome where it is degraded together with the autophagosome cargo. ATG8 lipidation is reversible since the covalent binding between ATG8 and PtdEth can be reverted by the proteolytic activity of ATG4

Autophagy is a catabolic process that mediates degradation of unnecessary or dysfunctional cellular components^2, 3^ (Figure 1). Through this mechanism cells eliminate damaged (and potentially dangerous) molecules or organelles, thus ensuring maintenance of cellular homeostasis. However, autophagy is also used as a cellular strategy to overcome intracellular or environmental stress, including nutrient deprivation, hypoxia and drugs effect.^4, 5, 6^ Degradation of cellular components may be an alternative mechanism to provide energy supplies and basic metabolites to cells.

This dual function makes autophagy a ‘Janus-faced' player in cancer progression. On the one side autophagy plays cancer-suppressive functions by eliminating potentially harmful components; on the other side it helps cells to overcome the stressful conditions that they undergo during cancer progression.^7^

**Figure 2:**
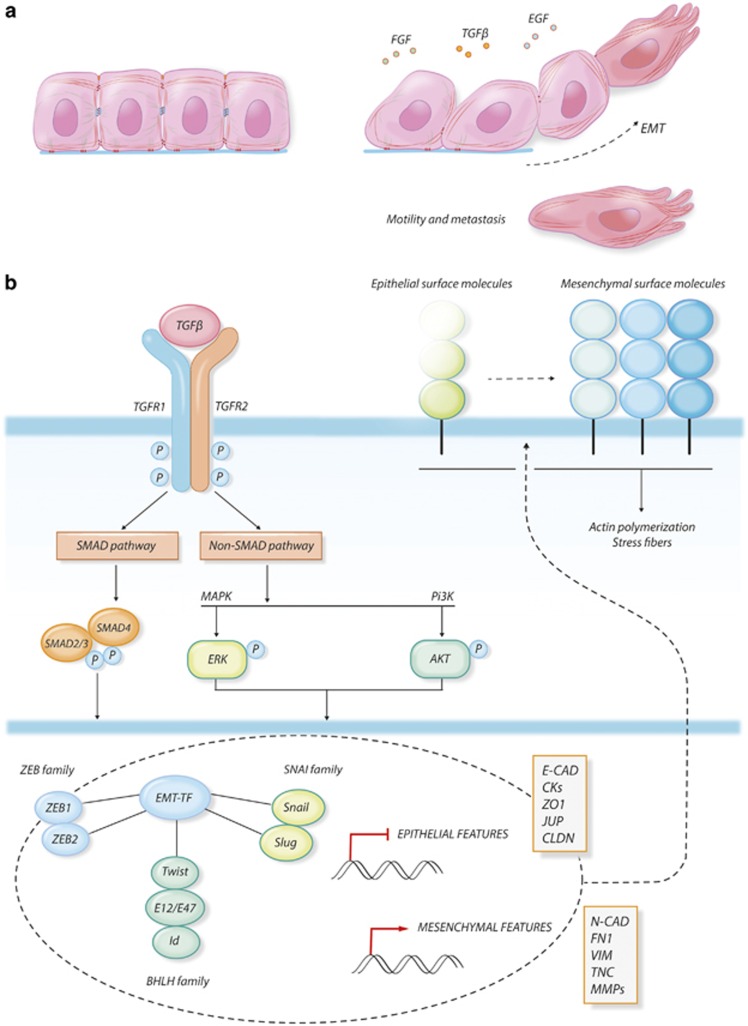
EMT. (**a**) Morphological changes in epithelial cancer cells undergoing EMT under growth factors stimulation. EMT is triggered by an interplay between different extracellular signals. Besides TGF*β*, the master regulator of EMT, other soluble growth factors like FGF and EGF can concur to activate EMT, which binding to their specific receptors and activating a cascade of intracellular mediators. (**b**) Schematization of TGF*β* signaling pathways driving EMT in cancer. The TGF*β-*activated receptors phosphorylate SMAD2/3, priming their dimerization with SMAD4, their translocation into the nucleus and their transcriptional activity. In addition to the SMAD-dependent pathway, TGF*β* can directly activate both the MAPK and the PI3K-class I pathways and their respective signaling cascades. All these pathways converge on the regulation of a specific network of transcription factors (TFs). SNAIL, SLUG, ZEB1 and 2, the bHLH transcription factor TWIST, E-proteins (E12/E47) and their repressors Ids are among the most characterized TFs in the regulation of EMT. Once activated, these transcription factors mediate the complex changes in gene expression profile that underlines EMT. The ability of the cells to interact with the surrounding microenvironment has to be completely re-designed during EMT, which is obtained by a complete reorganization of the adhesion molecules profiles on the cell membrane. Epithelial adhesion molecules that mediate the rigid cell–cell junctions are displaced by the cell membrane (either by transcriptional repression or by protein degradation) and are substituted by mesenchymal surface proteins. This process influences cytoskeleton dynamics, leading to actin polymerization and the formation of stress fibers

The epithelial to mesenchymal transition (EMT) is a biological process that allows epithelial cells to transiently assume mesenchymal features by undergoing profound molecular and biochemical changes^8^ (Figure 2). As a consequence of this process, epithelial cells shed their differentiated characteristics, including cell–cell adhesion, polarity and lack of motility to acquire more immature features, including high cellular plasticity, motility, invasiveness and resistance to apoptosis.^8, 9, 10, 11^ The EMT process and its reverse, the mesenchymal–epithelial transition, are leading mechanisms during embryonic development, when the correct equilibrium between these phenomena regulates the morphogenesis of organs and tissues.^12^ EMT is also the leading biological process of metastasis during cancer progression since it confers to cancer cells the ability to move from the original site to colonize adjacent or distant sites.^10, 11, 12, 13, 14^ Even though this process requires a precise regulation of gene expression and activation of specific signaling pathways, EMT is mainly determined by the morphological reprogramming of cellular architecture, which is guided by changes in the interaction properties of the cells with the surrounding microenvironment and is supported by a profound reorganization of the cell cytoskeleton.^15, 16, 17, 18^

Autophagy and EMT have been regarded for long time as too distant to be connected. However, recent observations indicate that these two important processes in cancer are linked in an intricate relationship. In this review we aim to revise the evidence of a possible interplay between autophagy and EMT in cancer.

## Evidence of Autophagy and EMT Functional Interaction

Several works reported a direct effect of autophagy on EMT regulation in cancer cells. In accordance with its dual role in cancer, the effect of autophagy on EMT appears controversial and likely dependent on the cellular type and/or on stage of tumor progression.

For its effect of improving resistance to unfavorable conditions, autophagy represents a powerful survival strategy for cells that are exposed to cell-intrinsic or environmental stressful stimuli. Indeed, several works show that defects in the autophagic machinery restrain dissemination and metastatic spreading of cancer.^19, 20, 21, 22, 23^ During metastatic spreading reorganization of the interaction properties and loss of the adhesion with the extracellular matrix leave cells without an effective anchorage and induce the activation of cell death pathways. The execution of the EMT program concurs to these massive cellular changes, promoting the exposure of cancer cells to potent demise stimuli. Autophagy induces resistance to cell death, providing a survival strategy for cells that are spreading outside the tumor mass. Fung and colleagues show that RNA interference-mediated suppression of ATG factors inhibits detachment-induced autophagy and enhances apoptosis in non-tumoral and primary mammary epithelial cells. The same authors also demonstrate that stable silencing of ATG5 or ATG7 enhances apoptosis and inhibits luminal colonization in a 3D culture model of MCF-10A breast cancer cells.^24^ Autophagy activation is also a general mechanism that cancer cells use to overcome the potential stress induced by hyper-activated tyrosine kinases in the absence of their key adaptor proteins tethering partners.^25, 26^ Sandilans and colleagues show that perturbations of the Integrin signaling through the FAK/Src axis, lead to the selective targeting of hyper-activated Src to autophagy degradation. Activation of autophagy is required for survival and protects cells toward the potential damages induced by uncontrolled Src activity.

In accordance with these observations, it has been recently reported that induction of autophagy is required for pulmonary metastasization of hepatocellullar carcinoma (HCC) cells. Stable silencing of the autophagic factors BECLIN1 (BCN1) and ATG5 in HCC cells impairs incidence of pulmonary metastases in an orthotopic mouse model. Autophagy inhibition does not affect migration or invasiveness capacity of HCC cells but reduces cell death resistance and lung tissue colonization.^27^ Also, N-myc downstream regulated 1 (NDRG1), known for its role of metastasis suppressor, has been shown to inhibit stress-induced autophagic responses, suggesting that its anti-metastatic activity could rely on its ability to prevent pro-survival autophagy activation in cancer cells.^28^

Since EMT promotes tumor metastasis and autophagy supports cell viability during the metastatic spreading, it is not surprising that EMT may promote autophagy in cancer cells. Autophagy is also a strategy used by cancer cells to promote immune surveillance escape and resistance to cytotoxic T-lymphocytes. Akalay and colleagues showed that acquisition of the EMT phenotype in MCF7 breast cancer cells is associated with attenuation in the formation of CTL-mediated immunologic synapse, increased autophagy and resistance to immune cells. Silencing of BCN1 and inhibition of the autophagic machinery restore susceptibility to T-cell cytotoxicity, suggesting that autophagy has a relevant function in helping EMT cancer cells to overcome the hostility of immune system during spreading.^29^

Resistance to apoptosis induced by changes in extracellular adhesion properties and the escape of the immune surveillance are major events for successful tumor metastatic spreading.

While EMT requires autophagy to support viability of potentially metastatic cancer cells, a number of additional evidence indicates that autophagy acts to prevent EMT and that the activation of the autophagic machinery may determine reversion of the EMT phenotype in cancer cells.^30, 31, 32, 33, 34^ It has been shown that induction of autophagy by nutrient deprivation or mTOR pathway inhibition leads to reduced migration and invasion in glioblastoma cells,^33^ while autophagy impairment determined by silencing of ATG5, ATG7 or BCN1 results in an increment of cell motility and invasiveness. Activation of the autophagic process leads to reduced stability and consequent downregulation of SNAIL and SLUG, two of the major transcription factors of the EMT process. This change in the transcriptional program determines re-expression of adhesion molecules and reversion of the EMT phenotype. Cadherin 6 (CDH6), a TGF*β*-target gene in the EMT process, is a major switch of the transition from the epithelial to the mesenchymal phenotype and a marker of metastatic potential in thyroid cancer. We recently identified GABARAP as the interactor of the cytoplasmic domain of CDH6. We showed that CDH6 silencing reverts the EMT phenotype by reducing proliferation and migration of thyroid cancer cells. The effect of CDH6 silencing is accompanied by induction of autophagy and alteration of mitochondrial dynamics.^34^

The death effector domain-containing DNA-binding protein (DEDD) is a key molecule for cell death signaling receptors. Lv and colleagues recently reported that the expression of DEDD attenuates EMT, acting as the tumor suppressor signal. These authors showed that DEDD physically interacts with the autophagic controlling complex PI3K/BCN1, leading to the autophagy-mediated lysosomal degradation of SNAIL and TWIST and consequentially to the attenuation of the EMT phenotype.^30^ An indirect evidence of the negative crosstalk between autophagy and EMT is represented by the observation that several anticancer compounds induce autophagy while inhibiting EMT in cancer cells.^35, 36, 37^

Selective degradation of specific EMT proteins seems to be the major molecular mechanism through which autophagy controls EMT. TWIST1 is a helix–loop–helix transcription factor that together with SNAIL and SLUG dictates activation of EMT.^38, 39^ Autophagy deficiency in squamous cell carcinoma (SCC) and melanoma results in stabilization and upregulation of TWIST1, which, in turn, induces the activation of EMT *in vitro* and promotes tumor growth and metastasization in mice.^31^ TWIST1 stabilization in autophagy-deficient cancer cells is mediated by the accumulation of sequestome-1 (SQSTM1/p62), an ubiquitin-binding protein which is a target of cargo-selective autophagy. SQSTM1/p62 binds to TWIST1 and prevents its degradation through either proteasome or autophagosome. As well in non-tumoral and cancer epithelial cells TGF*β* or other EMT inducing growth factors induce accumulation of SQSTM1/p62 that in turns stabilizes the TFG*β* effector SMAD4 and TWIST1 leading to changes in the expression pattern of junctional proteins.^40^ In another work, Grassi *et al.*^41^ using liver specific autophagy-deficient mice (Alb-Cre;ATG7fl/fl), show that autophagy degrades SNAIL in a p62/SQSTM1-dependent manner, restraining EMT and migration in hepatocytes.

The negative effect of autophagy on the acquisition of the EMT phenotype is also supported by the observation that lysosomal dysfunction leads to enhanced EMT. Lysosomal dysfunction induced by V-ATPase inhibition in podocytes causes deficiency of the autophagic flux and results in decreased expression of epithelial markers (like P-Cadherin and ZO1) and increased expression of mesenchymal proteins (including FSP-1 and a-SMA). Also in this case, SQSTM1/p62 seems to be the functional link between EMT and autophagy since its accumulation due to autophagy inhibition is functional for the acquisition of the EMT phenotype.^32^

Taken together these observations define a complex web of interactions between autophagy and EMT. On the one side, cells that underwent EMT require autophagy activation to survive during the metastatic spreading. On the other side autophagy, acting as oncosuppressive signal, tends to inhibit the early phases of metastasization, contrasting the activation of the EMT mainly by selectively destabilizing crucial mediators of this process.

## Shared Signaling Pathways and Molecular Mediators

**Figure 3:**
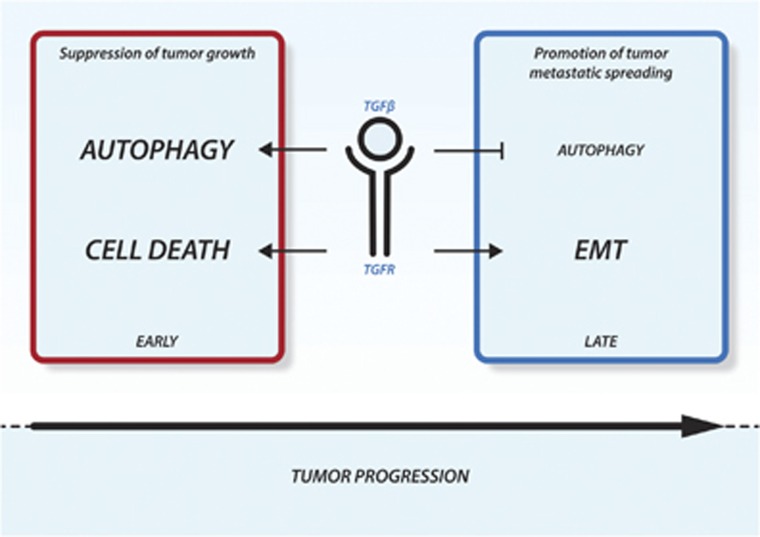
TGF*β* signaling dual effects in cancer. Schematic representation of the TGF*β* effect during cancer development and progression. In the early phases TGF*β* promotes autophagy and cell death suppressing tumor growth. Later, when tumor has settled TGF*β* restrains autophagy and induces EMT, promoting metastatic spreading

**Figure 4:**
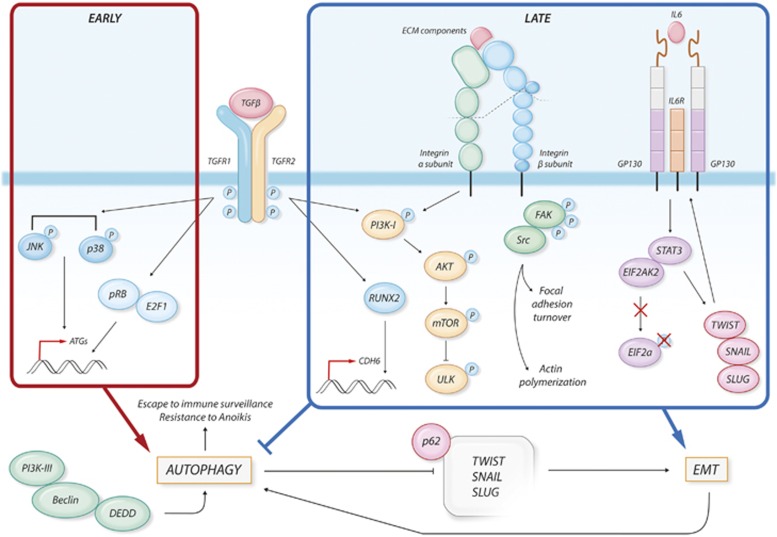
Signaling pathways at the crossroad between autophagy and EMT. During cancer progression, TGF*β* activates several pathways. In the early phases of tumor development, TGF*β* promotes the expression of pro-autophagic genes (ATGs) through the activation of the p38 and JNK pathways and through the induction of pRB/E2F1 transcriptional activity. On the other hand, during later phases of tumor progression, TGF*β-*mediated activation of the PI3K–AKT–mTOR pathway has been linked to promotion of EMT and to inhibition of autophagy through inhibition of ULK1. During EMT, TGF*β* also induces the expression of the mesenchymal marker CDH6, through the activity of the RUNX2 transcription factor. Changes in the ECM interaction properties of the cells lead to Integrin activation. the PI3K–AKT–mTOR and FAK–SRC axis are activated by Integrins and mediate the translation of mechanical and environmental stimuli within the cells. The FAK–Src pathway leads to FA turnover and actin polymerization, promoting the acquisition of a mesenchymal phenotype. IL-6 overexpression results in the activation of STAT3 that is able to sequestrate EIF2AK2, not allowing the phosphorylation of the pro-autophagic factor eIF2*α*. Moreover, STAT3 induces the expression of the EMT-promoting transcription factors TWIST, SNAIL and SLUG, also stimulating an autocrine signal through the upregulation of IL-6. Autophagy and EMT can also regulate each other. DEDD interacts with the autophagic-promoting complex PI3K-III/BCN1, leading to autophagy-mediated degradation of TWIST, SNAIL and SLUG thus attenuating EMT. In turn, EMT can enhance autophagy to help survive stressful conditions, escape immune surveillance and overcome cell death

Autophagy and EMT are both complex biological processes whose activation is tuned by an intricate web of molecular signals. Thus, it is not surprising that autophagy and EMT share common molecular mediators. The signaling pathways that regulate autophagy and those that control EMT have been extensively described in other reviews.^42, 43, 44, 45, 46, 47^ In this section we intend to summarize some of the evidence supporting a common regulation of these two processes (Figures 3 and 4).

### TGF*β*

TGF*β* is a multifunctional cytokine engaged in the regulation of multiple cellular functions.^48^ TGF*β* signaling generally plays oncosuppressive functions, repressing cell growth and inducing apoptosis. The inactivation of this pathway has been extensively linked to tumorigenesis. On the contrary, TGF*β* is known to be the most powerful activator of EMT during tumor progression (Figure 3). A TGF*β* ligand initiates signaling by binding to and bringing together type I and type II serine/threonine kinases receptor on the cell surface. This allows receptor II to phosphorylate the receptor I kinase domain, which then propagates the signal within the cells. The most relevant targets of TGF*β* receptors are the SMAD proteins. Once activated, SMAD proteins form heteromeric complexes that are translocated into the nucleus and, in conjunction with other nuclear cofactors, regulate the transcription of target genes.^48^ During EMT, TGF*β* signaling activates the expression of a panel of transcription factors like SNAIL, SLUG and TWIST1, which in turn orchestrate the Cadherin switch responsible for the changes in the adhesion properties of the EMT cells. We have already discussed how the destabilization of these TGF*β*-upregulated transcription factors is a mechanism by which autophagy reverts the EMT phenotype in cancer cells. Besides SMAD activation, TGF*β* signal is transduced within the cells through the activation of other non-canonical pathways. The p38/JNK, the PI3K/AKT/mTOR and MAPK signaling cascade are all activated by TGF*β* and somehow involved in the complex regulation of EMT.

Autophagy seems to be among the wide plethora of cellular processes under TGF*β* regulation. Several evidence indicates that TGF*β* activates autophagy in both normal and cancer cells.^49, 50, 51, 52, 53^ Kiyono *et al.*^49^ showed that TGF*β* treatment of HuH7 hepatocellular carcinoma cell line resulted in an early and strong activation of autophagy marked by the increased expression of pro-autophagic genes (BCN1, ATG5 and ATG7), the conversion of LC3 and the accumulation of autophagosomes. This TGF*β* effect is only in part mediated by the activation of the SMAD proteins and significantly relies on the activation of p38/JNK pathway. Indeed, silencing of JNK or its chemical inhibition attenuates the TGF*β*-mediated induction of autophagy and a failure of TGF*β* to induce the expression of ATG5. The activation of autophagy anticipates and is required for the TGF*β* induction of apoptosis since silencing of the pro-autophagic genes attenuates the TGF*β*-mediated growth inhibition and the induction of pro-apoptotic genes. In another work, Korah *et al.*^51^ reported that TGF*β*-dependent induction of autophagy is mediated by the activation of the pRB/E2F1 axis.^51^ Silencing E2F1 impairs the ability of TGF*β* to transcriptionally induce the expression of pro-autophagic genes. The same authors in a previous work reported that activation of the pRB/E2F1 axis is critical for the induction of multiple TGF*β*-targeted genes involved in tumor suppression, leading to apoptosis and cell growth inhibition.^54^ Based on this observation, it appears that, in cancer cells, TGF*β* activation of autophagy is functionally linked to the TGF*β*-dependent tumor suppressive effects. We recently showed that TGF*β* treatment in normal and thyroid cancer cell lines induces EMT through the activation of RUNX2 and its target CDH6.^55, 56, 57^ CDH6 silencing reverts the EMT phenotype and restrains migration and invasiveness in thyroid cancer cells. Noticeably, we demonstrated that CDH6 interacts with GABARAP and that CDH6 silencing also induces a massive activation of autophagy.^34^ Even if further experiments are needed, these evidence suggests that autophagy may negatively control EMT and that common signaling pathways control the dynamics of both processes. Furthermore, these observations identify CDH6 as a necessary mediator of the interplay between autophagy and EMT, uncovering a previously unknown function of Cadherins. Indeed, the interaction of CDH6 with the autophagic machinery is mediated by a structural epitope containing four sequential LIR domains that are strongly conserved in the cytoplasmic domain of the other Cadherins including E-CAD and N-CAD.

The activation of autophagy observed upon silencing of CDH6 is associated with a profound repression of AKT phosphorylation. The PI3K-I/AKT/mTOR is another non-SMAD pathway that can be activated in response to TGF*β.*^48^ The activation of this pathway has been mainly linked to the EMT-promoting effect of TGF*β*. mTOR is the catalytic subunit of two enzymatic complexes named mTORC1 and mTORC2 both of which may be activated by TGF*β* signaling.^58^ During EMT, TGF*β* promotes the increase of cell size, which is necessary to sustain cell migration and invasiveness.^59, 60^ This effect is mediated by mTORC1 which phosphorylates and activates the S6 kinase 1 (S6K1) and the eukaryotic initiation factor 4E-binding protein 1 (4E-BP1), both of which are direct regulators of translation initiation.^61, 62^ TGF*β* also induces mTORC2 kinase activity, which is required during the later phase to complete EMT. Loss of mTORC2 activity blocks cancer cell dissemination and metastasis formation in mouse models.^63^ In particular, mTORC2 phosphorylates and activates both RAC1 and PKCa both of which partake to the profound cytoskeleton reorganization that accompanies and sustains the morphological and functional changes of the cells during EMT.^64^ Furthermore, mTORC2 promotes cell invasiveness through the SNAIL-dependent upregulation of MMP9.^65^ Noticeably, the activation of the PI3K/AKT/mTOR pathway is the major inhibitory signal of autophagy.^58^ When activated, the mTORC1 complex leads to the phosphorylation and consequential inactivation of the serine/threonine UNC-51-like kinase (ULK1) that regulates the formation of autophagophores. The block of ULK1 activity results in the inhibition of the entire autophagic process.^66^ Thus, while promoting EMT through both SMAD-dependent and independent pathways, TGF*β* may restrain autophagy by mTORC1 mediated ULK1 inhibition.

Even if experimental evidence is still limited, it is reasonable to speculate that TGF*β* is able to trigger both pro-autophagic and anti-autophagic signals and the choice may be dependent on cellular context and on the cancer progression phase. In the early phases of tumor formation TGF*β* promotes autophagy as part of the TGF*β*-dependent tumor suppressive program. Later in tumor progression, TGF*β* restrains autophagy while inducing EMT and promoting metastatic spreading of cancer cells (Figures 3 and 4).

### STAT3

As discussed above, several transcription factors are involved in EMT. Recently a relevant role of the transcription factor Signal Transducer and Activator of Transcription 3 (STAT3) in promoting EMT has been proposed.^67^ The aberrant expression of interleukin 6 (IL-6) and its activation of STAT3 have been correlated with the development and progression of carcinomas^68^ and in particular with increased metastatic potential and poor outcome in epithelial tumors.^69^ IL-6-STAT3 signaling promotes the initiation of EMT and the acquisition of mesenchymal features in breast and head and neck cancers, by inducing the expression of Twist and Snail. In turn, TWIST upregulates the production of IL-6, leading to autocrine activation of STAT3.^70^ Saitoh and colleagues showed that STAT3 acts as molecular mediator of the synergism between TGF*β* and RAS signals during EMT. In particular, these authors observed that upon TGF*β* stimulation, STAT3 dissociates from Protein Inhibitor of Activated STAT3 (PIAS3) and, in cooperation with the RAS signaling, induces the expression of SNAIL and the progression of the EMT process.^71^ Intriguingly, evidence for a non-transcriptional role of STAT3 in controlling cytoskeleton organization has been proposed. In particular, STAT3 has been shown to interact with the microtubule (MT)-destabilizing protein STATHMIN, inhibiting its function. The expression of STAT3 is required for the stabilization of microtubules and cell migration.^72^

STAT3 is one of the major signaling pathways activated in response to stress, but it is also known to control autophagy in various ways.^73^ Cytoplasmic STAT3 constitutively inhibits autophagy in a transcription-independent manner. Shen and colleagues, in a screening designed to identify novel inducers of autophagy, showed that inhibitors of STAT3 induced a strong activation of autophagy.^74^ These authors show that the SH2 domain of STAT3 mediates its interaction with the catalytic domain of the eIF2*α* kinase 2 (EIF2AK2), also known as protein kinase R (PKR), inhibiting its function. This inhibition blocks the phosphorylation of eIF2*α* and its pro-autophagic function, thus restraining the autophagic flux. Beside, cytoplasmic STAT3 inhibits autophagy also by interacting with other autophagy-related signaling molecules such as Forkhead box protein O1 (FOXO1) and FOXO3.^75^ Nuclear STAT3 also controls autophagy by fine tuning several autophagy-related genes. In this context, STAT3 may promote as well as restrain autophagy depending on its targets. Nuclear STAT3 executes antiautophagic functions by upregulating negative regulators of autophagy such as BCL2, BCL2L1, MCL1, PIK3R1/p55*α* and PIK3R1/p50*α*.^76, 77, 78^ Furthermore, STAT3 disrupts the formation of the pro-autophagic BCN1/PI3K-III complex by recruiting histone deacetylase 3 (HDAC3) to the promoter of BECN1 and repressing its expression. During EMT Integrin profiles and Integrin-ECM contacts undergo profound reorganization (Figure 4).

### Integrins and focal adhesion

The diversity in binding affinities of different Integrins enables cells to respond to a vast variety of stimuli and activate intracellular cascades that cooperate to initiation and completion of EMT.^79^ Noticeably, stimulation of Integrins by ECM components leads to the activation of the Integrin-linked kinase (ILK) that promotes the phosphorylation of AKT and the consequential activation of the AKT–mTOR cascade, which we have already discussed to be the major inhibitory signal of autophagy.^80^

Recently, it has been shown that inhibition of Integrins in glioma cells causes massive cell detachment and death due to the activation of an atypical anoikis. This effect was associated with a strong inhibition of the TGF*β* pathway and the parallel activation of autophagy, again suggesting an intimate and reciprocal interaction between EMT and autophagy controlling mechanisms.^81, 82^

Focal adhesions (FAs) are Integrin-based multiprotein complexes that link ECM to intracellular actin cytoskeleton and are responsible for the translation of mechanical forces and other regulatory stimuli within the cells. FAs are highly dynamic structure that may rapidly assemble and disassemble at the edge of moving cells, controlling migration.^83, 84, 85, 86, 87, 88^ FAs appear to be a central hub of the crosstalk between autophagy and EMT. Several papers demonstrate that autophagy participate to FA turnover affecting cell migration.^81, 89, 90^ As reciprocal feedback, FAs control autophagy, mainly through the FAK–Src signaling pathway.^25, 26^ By selective targeting FA proteins to autophagosome degradation, autophagy promotes FA disassembly and sustains cell migration. During this process autophagosomes localize to dynamic FAs and selectively sequestrate FA components (paxilin, vinculin, etc.) into autophagosomes, promoting local FA destabilization and disassembly.^89, 90^ However, depletion of ATG proteins not only reduces FAs disassembly but also negatively affect their assembly, suggesting that autophagy participates also to stabilization of FAs, likely through more indirect mechanisms.^89^ Indeed, Tuloup-Minguez *et al.*^81^ showed that autophagy selectively targets and degrades Integrin *β*1, mitigating Integrins surface expression and inhibiting cell migration.^81^

As discussed above, impairment of FAK–Src signaling either by loss of function or by impaired FAK phosphorylation triggers autophagy activation.^25, 26^ Of note, the FAK–Src axis contributes to EMT by promoting the acquisition of a mesenchymal phenotype through different mechanisms.^83, 84, 85, 86, 87, 88^ Noticeably, in cancer cells overexpression of FAK mutants unable to be phosphorylated or pharmacological inhibition of FAK kinase activity restrain EMT by blocking the SRC-induced internalization of E-cadherin, increasing cell adhesion strength.^84, 85^ Taken together, this evidence indicates that FAK inhibition both restrains EMT and promotes selective autophagy. To establish whether a functional link between these two events exists, further experimental proofs are still required. However, the centrality of FAs and FAK–Src axis in the regulation of these processes highlights the relevance of mechanical stimuli and cell structure regulation at the crossroad between autophagy and EMT (Figure 4).

## Autophagy and EMT in Cancer: Is it a Matter of Cytoskeleton and Mitochondria?

Cytoskeleton remodeling is crucial to accomplish EMT.^91, 92^ Changes in the adhesion molecule profile (like the Cadherin switch or FAs remodeling) during EMT determine the activation of actin polymerization and the formation of non-polarized thick fibers named stress fibers.^93^ These cytoskeleton structures are necessary to support cell movement and to sustain mechanical stress imposed on cells by the loss of cell–cell and cell–ECM interactions.^94^ Cytoskeleton remodeling is not just the default consequence of EMT activation, but it plays a regulatory role in this process.^17, 95^ Depolymerization of the actin cytoskeleton reduces cell size, changes cell shape and reverses EMT phenotype in cancer cells.^17, 95, 96, 97, 98^ Furthermore, depolymerization of actin filaments induces the nucleus to cytoplasm translocation of SNAIL, leading to re-expression of E-CAD and inhibition of EMT.^17^

**Figure 5:**
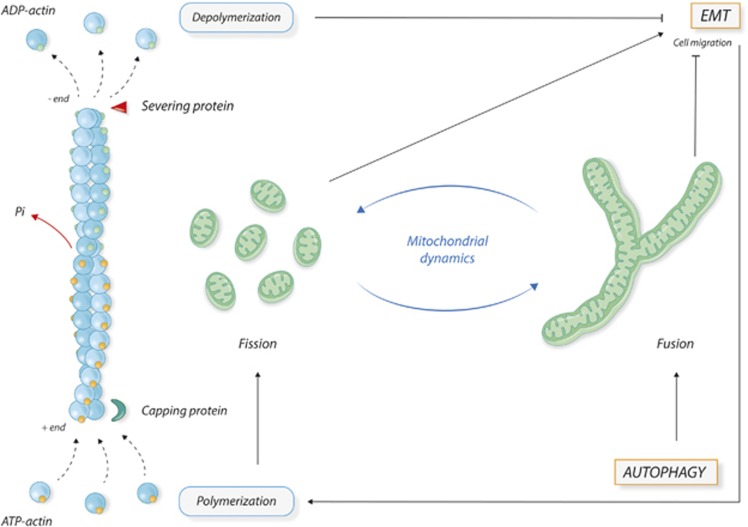
Schematic representation of the crosstalk between cytoskeleton and mitochondria and its possible role in the interplay between autophagy and EMT. Cytoskeleton and mitochondrial dynamics are in a tight relationship and their modifications play active roles in several cellular processes, among which EMT and autophagy. Depolymerization of actin cytoskeleton reverses EMT phenotype. On the other side, EMT activation leads to cytoskeleton polymerization and remodeling, which in turn supports mitochondrial fission, necessary to sustain cell migration and EMT process. Massive activation of autophagy induces mitochondrial fusion and the reconstitution of mitochondrial network, resulting in a reduced availability of free mitochondria, with a subsequent reduction of cell migration and EMT

Mitochondria are highly versatile organelles, which are prominent players of energy conversion and integrate a number of signaling pathways. Cytoskeleton and mitochondria are linked in a tight functional relationship, which is particularly relevant in the regulation of cell migration (Figure 5). Beside their morphological plasticity, mitochondria are characterized by the ability to move across the cells to specialized cellular sites where their energy support is majorly required. Movement and localization of mitochondria within the cells are determined by their interaction with the cytoskeleton.^97^ In particular, the interaction of mitochondria with microtubules and actin filaments carries mitochondria around the cells, ^98^ while the interaction with intermediate filaments serves to stop mitochondria in specialized cellular sites.^99, 100^ Accumulation of mitochondria below the cell membrane is necessary to promote the formation of filopodia and lamellipodia, cellular structure for motility during EMT.^101, 102^ Pharmacological inhibition of PI3K pathway in cancer cells promotes cell motility. This effect is mediated by the increased trafficking of energetically active mitochondria to the cortical cytoskeleton of tumor cells where they support FA turnover, membrane protrusion dynamics, migration and invasiveness.^103^

The functional versatility of mitochondria is dependent on their morphological and structural plasticity. Mitochondria fuse and fragment during cell life appearing either as short round-shaped units or as elongated branches forming a network.^104^ The morphological appearance of mitochondria in a cell is dependent on the balance between the two opposing processes of fission (fragmentation into single units) and fusion (melting of mitochondria into a well-structured network). Profound changes in the structure of mitochondrial network occur in cancer.

Increased mitochondrial fission and loss of mitochondrial network have been described as features of oncogenic transformation^105^ and increased cancer aggressiveness.^106, 107^ Furthermore, the GTPase DRP1, which drives mitochondrial fission, is strongly upregulated in metastatic as compared with non-metastatic cancer cells,^108, 109^ confirming that a fractioned network and single unit mitochondria are required during cancer progression.

In order to be moved around the cells, mitochondria need to be as single units, free from the tight network organization. Convincing evidence indicates that actin polymerization promotes mitochondrial fission.^110, 111, 112, 113, 114, 115, 116^ In addition, Xu *et al.*^117^ showed that activation of the EMT program by TGF*β* in A549 lung cancer cells leads to a significant increase in the overall mitochondrial content. Vice versa, actin-depolymerizing drugs have been shown to inhibit the recruitment of DRP1 to mitochondria and, as a consequence, to inhibit the reduction of mitochondrial length.

Also autophagy participates to the control of mitochondrial dynamics. Mitochondria can be degraded either through non-selective autophagy or through a cargo-selective specific autophagy called mitophagy.^118, 119, 120, 121, 122^ Mitophagy activation requires mitochondrial fission.^123, 124^ Upon fission, mitochondria expose a kind of ‘eat me' signal that targets mitochondria to autophagosomes for degradation. By contrast, the massive activation of unselective autophagy induces mitochondria fusion.^125, 126, 127^ Elongated fused mitochondria are spared from autophagy degradation and may help to optimize ATP production during starvation. Both high levels of mitophagy or unselective autophagy result in a reduced availability of free mitochondria within the cells with an impact on the formation of structures like filopodia and lamellipodia and thus a reduction of cell migratory capacity.

Based on these observations, it is tempting to speculate that the regulation of mitochondria and cytoskeleton dynamics and of their functional interplay represents a preferential and rapid way of interaction between EMT and autophagy (Figure 5). EMT activation changes the cell adhesion profile leading to cytoskeleton remodeling. Actin polymerization in turn promotes fragmentation and trafficking of mitochondria to the cortical regions where they provide energy supplies for motility membrane protrusions and cell migration. By contrast, activation of autophagy reduces the number of available free mitochondria counteracting the EMT phenotype.

Even if definitive evidence is still missing the first experimental indications supporting this model have recently begun to emerge.

Metabolic reprogramming is an important driver of cancer progression. Even if under extremely unfavorable energetic conditions, metastatic cancer cells manage to acquire highly energetically demanding features like migration and invasiveness. Mitochondria activity seems to be crucial in this process. Caino and colleagues showed that under nutrient deprivation condition, cancer cells preserve cytoskeletal dynamics and motility through the chaperone function of mitochondrial associated HSP90. Functional maintenance of HSP90 mitochondrial target proteins guaranties residual oxidative phosphorylation and ATP production preventing autophagy activation. Inhibition of autophagy restrains the sequestration and consequential inhibition of FAK by the ULK1/Atg13/FIP200 autophagic complex, supporting cell motility and migration.^128^

A recent work showed that the mitochondrial protein BNIP3 controls plasticity of actin cytoskeleton and cell motility in melanoma.^129^ BNIP3 is engaged in mitophagy since it mediates the interaction of mitochondria with the autophagosome ATG8 proteins (LC3 and GABARAP) to promote mitophagy. Ablation of BNIP3 induces structural changes in the adhesion properties of melanoma cells and a strong reorganization of the actin filaments. These changes determine the reduction of lamellipodia and filopodia formation and of the migratory capacity of melanoma cells. We recently found BNIP3 and BNIP3L (together with GABARAP) as CDH6 interactors in thyroid cancer cells. Noticeably, upon CDH6 silencing we observed a phenotype similar to the one showed by BNIP3 silenced melanoma cells. In particular, loss of CDH6 causes a profound reorganization of cytoskeleton with a marked reduction of cell surface protrusions and reversion of the EMT phenotype. This effect is accompanied by a massive activation of autophagy and by profound alterations of mitochondria that in the absence of CDH6 fuse into a well-organized network, revealing an unexpected link between Cadherins and mitochondrial dynamic.^34^

## Conclusion

During cancer progression, a complex and non-linear relationship links EMT and autophagy. The interplay between these biological processes is influenced by several aspects and functionally evolves throughout the different phases of tumor development and progression. This complexity is reflected by the intricate web of regulatory signaling pathways that converge on the regulation of EMT and autophagy and that may alter the reciprocal equilibrium between these two processes.

The tight relationship between cytoskeleton and mitochondria and their importance in regulating both these processes is emerging as a novel layer of reciprocal regulation between EMT and autophagy that deserves further investigations.

Furthermore, the recently observed ability of membrane proteins like Integrins or Cadherin to affect autophagy underlines the complexity of these proteins functions, which may not any longer being considered merely structural proteins.
